# The therapeutic value of ipsilateral supraclavicular lymphadenectomy in locally advanced breast cancer: A review

**DOI:** 10.1097/MD.0000000000039340

**Published:** 2024-08-16

**Authors:** Sen Li, Yingfang Shi, Zhichun Wang

**Affiliations:** a Breast Surgery, Jiujiang First People’s Hospital, Jiujiang, Jiangxi Province, China.

**Keywords:** breast cancer, ipsilateral supraclavicular lymphadenectomy, locally advanced

## Abstract

The treatment of isolated ipsilateral supraclavicular lymph node metastasis for locally advanced breast cancer has always been a controversial issue for breast surgeons. However, with the further understanding of the metastasis and treatment of breast cancer, it is now considered to be a locally advanced disease, and there is a new debate on the treatment of isolated ipsilateral supraclavicular lymph node metastasis. The author reviewed the relevant literature and briefly discussed the clinical significance of supraclavicular lymph node resection in patients with locally advanced breast cancer presenting with isolated ipsilateral supraclavicular lymph node metastasis.

## 1. Introduction

Breast cancer is a highly heterogeneous malignant tumor, showing a gradually increasing incidence trend in our country, which seriously threatens women’s health.^[1]^ The modes of metastasis of breast cancer include local extension, blood supply metastasis and lymphatic metastasis.^[2]^ In the lymphatic metastasis pathway, tumor cells can invade the ipsilateral axillary lymph nodes through the lateral border lymphatic vessels of the pectoralis major muscle, and then invade the subclavian lymph nodes and then the supraclavicular lymph nodes. In addition, the medial lymphatic vessels can invade the parasternal lymph nodes along the perforating branches of the internal mammary vessels, thereby invading the supraclavicular lymph nodes.^[2, 3]^ Clinically, the incidence of isolated ipsilateral supraclavicular lymph node metastasis (ISLM) is low, and breast cancer patients with ISLM account for about 1% to 4.3%.^[4,5]^ Due to its low incidence, there has been a lack of prospective, large clinical randomized controlled trials on the choice of treatment methods for ISLM. At present, the common treatment principle is to use adjuvant systemic therapy, including chemotherapy, targeted therapy, endocrine therapy, etc, on the basis of surgical treatment of the primary tumor. Radiotherapy is the main local treatment for the supraclavicular region, and surgical treatment of local supraclavicular lymph node resection has not been recommended as the standard treatment for ISLM. Whether to perform supraclavicular lymph node dissection for ISLM patients has become a difficult choice for breast surgeons.

## 2. Methods

### 2.1. Search strategy

A comprehensive literature search was conducted across multiple online databases to identify relevant studies. Using Medline, Google Scholar, Web of Science, and PubMed, the search utilized MESH terms including “ipsilateral supraclavicular lymphadenectomy,” “breast cancer,” “locally advanced,” and “ISLM.” The search, conducted in January 2024, was not restricted by publication date.

### 2.2. Eligibility criteria

The inclusion criteria encompassed all prior original studies, regardless of study design, that examined ISLM in locally advanced breast cancer. Excluded from the analysis were editorials, reviews, and opinion articles.

### 2.3. Data extraction

The data extracted from each study included surgical approach, postoperative follow-up duration, survival-related data. Additionally, author names, publication dates, sample sizes, and study designs were also extracted and compiled into Table 1.

**Table 1:** Abstract of review papers.

Study number	First author	Year	Study design	Sample size	Summary of results
1^[6]^	Tezuka K	2011	Case report	1	No signs of tumor recurrence or metastasis were found 7 years after operation.
2^[7]^	Chen SC	2006	Retrospective study	Of 63 ISLM patients, 19 patients had received fine-needle aspiration cytological analysis only, 15 had excisional biopsies under local anesthesia, and 29 had more radical surgery (supraclavicular level IV and V lymph node dissection).	The 5-year OS of patients who underwent level IV and V neck lymph node dissection was significantly better than that of patients who underwent fine needle aspiration biopsy alone (42.4% vs 16.3%, *P* = .0327).
3^[8]^	Chen S	2010	Retrospective study	Of 127 ISLM patients, 49 patients underwent neck lymph node dissection of level IV and some levels III and V. 78 patients underwent fine-needle aspiration biopsy without local surgery.	With a median follow-up of 6.9 years, the 5- and 10-year OS rates were 30.6% and 16.1%, respectively, which were significantly higher than those of patients without cervical lymph node dissection (14.9% and 4.7%). The hazard ratio (HR) was 1.72 (95% CI 1.17–2.52).
4^[9]^	Chang XZ	2013	Retrospective study	Of 29 ISLM patients, 13 patients underwent supraclavicular lymph node dissection. Sixteen patients underwent received full-dose radiation therapy.	After a 47-month follow-up, the 5-year OS rates were 46.2% with supraclavicular lymphadenectomy and 37.5% with full-dose radiotherapy, while the 3-year DMFS rates were 46.2% and 31.3%, respectively, with no significant differences observed between the groups (*P* = .492 for OS and *P* = .107 for DMFS).
5^[10]^	Zhang W	2017	Retrospective study	Of 90 ISLM patients, 34 patients underwent supraclavicular lymph node dissection. 56 patients underwent received full-dose radiation therapy.	Survival analysis showed that there was no significant difference in overall survival rate and recurrence-free survival rate between the surgery group and the control group (*P* = .359, *P* = .246).
6^[11]^	Pan H	2021	Population-based cohort study	Of the 2033 patients, 346 patients had distant lymph node metastases, 212 patients had ISLM, and 1475 patients had distant metastases.	Locoregional treatment was associated with improved survival for non-distant metastases cases.

### 2.4. Ethical approval

Given that this was a literature review, ethical approval was deemed unnecessary.

## 3. Ipsilateral supraclavicular lymphadenectomy

### 3.1. History and Evolution

Surgeons’ understanding of supraclavicular lymph node metastasis in breast cancer can be traced back to 1907, when Halsted published a clinical study on 44 breast cancer patients with histologically confirmed supraclavicular lymph node involvement, and only 2 of the 44 patients (5%) survived for >5 years.^[12]^ In 1956, Haagensen^[13]^ reported that breast cancer patients with supraclavicular lymph node metastasis could not achieve a 5-year clinical cure, and proposed that surgical treatment was not recommended for patients with supraclavicular lymph node metastasis. ISLM is regarded as a sign of poor prognosis. Cancer cells can spread from the supraclavicular lymph node to the blood through the right lymphatic vessel or thoracic duct, and then to the distant metastasis. Most patients will have distant metastasis within 1 year, and the median survival time is <20 months.^[5]^

Based on the understanding of the poor prognosis of ISLM, ISLM was defined as distant metastasis – M1 stage in the fifth edition of the American Joint Committee on Cancer (AJCC) guidelines in 1987. The change of ISLM staging leads to clinicians’ pessimistic judgment on the prognosis of these patients and negative choice of treatment methods. At this time, the main treatment for ISLM patients is palliative care for palliative purposes. However, Brito et al^[14]^ reported in 2001 that 70 breast cancer patients with ISLM received comprehensive treatment, including surgical treatment, chemotherapy, and radiotherapy, etc. The results showed that the prognosis of breast cancer patients with ISLM was much better than that of breast cancer patients with metastasis to other distant organs. Therefore, it is proposed that breast cancer patients with ISLM can be completely cured under certain conditions. Thus, Brito advocates aggressive treatment of patients with tumor spread confined to the ipsilateral supraclavicular lymph nodes and reclassification of this group to stage III in the TNM classification. In 2003, the AJCC revised the TNM staging of breast cancer and classified ISLM as N3c, IIIC stage, which belongs to locally advanced stage. The new classification of ISLM was maintained in the 7th edition of AJCC guidelines in 2010.

The new understanding of ISLM in breast cancer has brought about clinical changes in the treatment of such patients, and more local treatments have been used for such patients, including local radiotherapy and local surgical treatment. Compared with surgical treatment, the efficacy of radiotherapy is more clear and less controversial.^[7, 15, 16]^ However, the surgical treatment plan is in an awkward position in the treatment of ISLM for breast cancer because of the special anatomical position of supraclavicular lymph nodes, the scope of lymph node dissection after surgery is vague, the difficulty and risk of surgery are great, there is the possibility of surgery-related complications, and the survival benefit brought by local surgery is unclear.^[17]^

### 3.2. Related research and progress

For a considerable period of time, supraclavicular lymph node dissection in ISLM breast cancer patients was regarded as a contraindication to surgery. However, in 2011, Tezuka^[6]^ reported a case of breast cancer patient with ISLM after modified radical mastectomy, and then the metastatic supraclavicular lymph nodes were removed by supraclavicular lymph node dissection. Chemotherapy was subsequently administered. Three years after the modified radical mastectomy, ISLM was found in the outpatient reexamination. No tumor remission was observed with oral antineoplastic drugs, and no metastasis was found at reexamination. Subsequent ipsilateral supraclavicular lymph node dissection was performed 1 year later, and no signs of tumor recurrence or metastasis were found 7 years after operation. Therefore, they proposed that after screening of relevant conditions, some patients can achieve satisfactory treatment results by supraclavicular lymph node dissection. Due to the low incidence of ISLM and medical ethical issues, there are relatively few studies on the effect of local surgery for breast cancer patients with ISLM, especially the lack of large randomized controlled clinical trials. However, some retrospective studies can also help us to gain something.

In 2006, Chang Gung Memorial Hospital conducted a retrospective study on the ISLM treatment of 3170 primary breast cancer patients in the 1990s.^[7]^ The results of this study showed that the incidence of ISLM in primary breast cancer patients was significantly lower than the local recurrence rate and distant metastasis rate, that is, 63 cases of ISLM occurred in all 3170 patients. Local recurrence and distant metastasis occurred in 151 and 599 patients, respectively. The prognosis of ISLM patients is worse than that of patients with subclavian lymph node metastasis. The overall survival (OS) of ISLM patients was better than that of patients with distant metastasis, and the prognosis of ISLM patients was not statistically different from that of patients with more than 9 positive axillary lymph nodes. The 5-year OS of patients who underwent level IV and V neck lymph node dissection was significantly better than that of patients who underwent fine needle aspiration biopsy alone (42.4% vs 16.3%, *P* = .0327).

In 2010, Chen^[8]^ reported the results of their retrospective study of 5409 cancer patients who underwent surgery, of which 127 (2.3%) patients developed ISLM. Of 127 ISLM patients with cancer, 49 patients underwent neck lymph node dissection of level IV and some levels III and V. 78 patients underwent fine-needle aspiration biopsy without local surgery. With a median follow-up of 6.9 years, the 5- and 10-year OS rates were 30.6% and 16.1%, respectively, which were significantly higher than those of patients without cervical lymph node dissection (14.9% and 4.7%). The hazard ratio (HR) was 1.72 (95% CI 1.17–2.52), and the survival prognosis of the neck lymph node dissection group was significantly better than that of the non-surgery group.

However, the results of some studies are inconsistent. In 2013, Chang et al^[9]^ reported their single-center retrospective study of cancer ISLM, which retrospectively analyzed 29 patients with ISLM without distant metastasis. All patients had undergone radical or modified radical mastectomy and subsequent systemic therapy. Thirteen of these patients underwent supraclavicular lymph node dissection, and the remaining 16 patients received full-dose radiation therapy. After a median follow-up of 47 months, 23 patients developed distant metastases. The 5-year OS was 46.2% with supraclavicular lymphadenectomy and 37.5% with full-dose radiotherapy. There was no significant difference in OS curve between the 2 groups (*P* = .492). The 3-year distant metastasis-free survival (DMFS) was 46.2% in the supraclavicular lymph node dissection group and 31.3% in the full-dose radiotherapy group. There was no significant difference in DMFS curve between the 2 groups (*P* = .107). Although the prognosis in the 2 groups was similar in the survival analysis, there were some differences, with 3 patients in the dissection group surviving >5 years without recurrence. However, no patients with 5-year DMFS existed in the radiation therapy group. Finally, the study suggested that supraclavicular lymph node dissection should be considered for ISLM in individual cases.

In the retrospective analysis reported by Zhang et al,^[10]^ a total of 90 breast cancer patients newly diagnosed with ISLM were included in the study. According to whether the patients underwent supraclavicular lymph node dissection, they were divided into the surgery group (34 cases) and the control group (56 cases). The median follow-up time was 85 months. During the follow-up, 32 patients had locoregional recurrence, 47 patients had distant metastasis, and 25 patients had both. Of the 32 patients with local recurrence, 11 were in the surgery group and 21 in the control group. Of the 47 patients with distant metastasis, 17 were in the surgery group and 30 in the control group. At the end of follow-up, 32 of the 90 patients died, 16 in the surgery group and 16 in the control group. Survival analysis showed that there was no significant difference in overall survival rate and recurrence-free survival rate between the surgery group and the control group (*P* = .359, *P* = .246). The researchers proposed that supraclavicular lymph node dissection may be an effective means of local control for estrogen receptor negative, progesterone receptor negative breast cancer patients with initial supraclavicular lymph node metastasis, but may be detrimental to the overall survival of human epidermal growth factor receptor-2 negative breast cancer patients with initial supraclavicular lymph node metastasis.

ISLM is a special form of locally advanced breast cancer, which has a low clinical incidence and poor prognosis. However, does its prognosis have any advantages over that of patients with distant lymph node metastasis without visceral metastasis? In the study published by Pan et al,^[11]^ the enrolled patients were divided into 3 groups: the distant lymph node metastasis group, the visceral metastasis group and the ISLM group. The primary end point of the study was OS, and the secondary end point was breast cancer specific survival rate (BCSS). There was no significant difference in OS (HR = 0.73, *P* = .09) or BCSS (HR = 0.81, *P* = .34) between the patients with distant lymph node metastasis and those with visceral metastasis, but there was a significant difference in OS (HR = 1.79, *P *< .001) and BCSS (HR = 1.99, *P *< .001) between the 2 groups. The survival prognosis is significantly worse. This study showed that the prognosis of ISLM patients was similar to that of patients with distant lymph node metastasis, and was significantly better than that of patients with visceral metastasis. Moreover, local treatment of ISLM for supraclavicular lymph nodes could improve survival benefit, even for cervical lymph nodes farther than the supraclavicular lymph nodes.

### 3.3. Prospects of biological mechanism

As a locally advanced state, systemic therapy is the first choice for ISLM patients according to relevant guidelines. Whether local surgical treatment can bring survival benefits is still unclear. Based on the satisfactory results of systemic treatment, local surgical treatment can be included in the eligible patients. For patients with poor systemic treatment, other systemic treatment is recommended instead of local surgical treatment. In recent years, the oligometastatic theory of breast cancer has been accepted by more and more clinicians, suggesting that not all ISLMs are consistent, and some patients may benefit from surgical treatment.^[18]^

Even though some preclinical studies have shown that resection of the primary tumor may cause distant metastasis of tumor cells.^[19–21]^ The postulated mechanisms include the increase of vascular endothelial viscosity between circulating tumor cells and metastatic target organs caused by surgical stress, tumor immunosuppression induced by surgical stress, surgical induced angiogenic switch or triggered immune cascade.^[22–24]^

In clinical practice, it can be noted that some malignant tumors may have a relatively durable stable stage before distant metastasis, suggesting that there may be a “gentle” period between the primary tumor and distant metastasis. In 1995, Weichselbau et al proposed the theory of oligometastasis for the first time. They believed that compared with extensive tumor metastasis, oligometastasis is a relatively early stage in the process of tumor metastasis, which is a transitional stage, in which most of the related genes that can stimulate rapid tumor growth are not fully expressed, and the related stimulating factors are not fully activated. The tumor shows mild aggressiveness, and the ability of widespread dissemination and metastasis has not yet been demonstrated. Local treatment at this stage can obtain great clinical value. The author believes that in terms of some clinical symptoms and prognosis, ISLM is similar to the “oligometastasis” state, which is an oligometastasis in a sense.

In the 3rd International Consensus Conference on Advanced Breast Cancer, oligometastases were defined as the presence of fewer than 5 metastatic lesions in <2 organs.^[25]^ Moreover, the 4th International Consensus Conference on Advanced Breast Cancer clearly suggested that oligometastatic lesions may achieve clinical benefits through active local treatment.^[26]^ In 2018, the 11th European Congress on Breast Cancer reported the research progress in the treatment of breast cancer oligometastases, which showed that after local active surgery and radiotherapy, the quality of life and OS of some patients with oligometastases including brain metastasis, bone metastasis, liver metastasis could be significantly improved.

The theory of oligometastatic breast cancer has inspired the treatment idea of ISLM. In the face of a small number of metastatic lymph nodes, the treatment strategy aiming at cure or long-term clinical remission is encouraged, and better clinical guidance can be obtained. In the face of extensive tumor metastasis, surgical treatment to control local progression can be used as a palliative and remission treatment to improve the quality of life of patients. The treatment strategy of oligometastasis is to optimize systemic treatment while emphasizing active local intervention. It is very important to enroll the appropriate patient population, therefore, the judgment of the eligible population is particularly important.

In addition to the oligometastatic theory of breast cancer, in clinical practice, patients who can achieve “complete response” of the tumor after systemic treatment often indicate a better prognosis. Therefore, it is theoretically feasible to achieve effective remission and stability in a certain period after systemic treatment, and to achieve “complete response” by local surgery. And should benefit patients; Due to the differences in biopsy pathological evaluation, gene mutation, tumor heterogeneity, clonal selection, tumor microenvironment heterogeneity, detection error and other factors, there are differences in pathological information between metastatic lesions and primary tumors in clinical practice.^[27–30]^ Through local resection, sufficient, complete and detailed pathological information can be obtained, which is conducive to accurately guiding the next treatment. Moreover, studies have confirmed that repeat biopsy can lead to changes in treatment strategies in 20% of patients.^[31]^

## 4. Conclusion

In current clinical practice, the regional management strategy for breast cancer patients with ISLM should comprehensively consider the efficacy of systemic therapy, the impact of staging information on subsequent treatment, and the efficacy and adverse reactions of different local regional treatments. Through reviewing relevant studies, we found that in certain specific ISLM breast cancer patients, undergoing supraclavicular lymph node dissection surgery may confer survival benefits. This subset of ISLM resembles a “oligometastatic” state, wherein in this relatively early transitional state, combined with specific breast cancer subtypes, patients post-surgery can pursue a state of cure.

Therefore, among breast cancer patients with ISLM, we believe that a subset of patients may derive relatively favorable survival benefits from proactive local surgical interventions following careful and meticulous screening. The focus of future research should be on how to ensure that the eligible population receives the corresponding treatment, incorporating patient demographics, tumor staging, molecular subtypes, comprehensive treatment strategies, and even relevant risk models established based on factors such as genetic risk scores. However, considering the individualized and precise nature of tumor treatment, more robust data and comprehensive clinical studies are still needed for exploration.

## Author contributions

**Conceptualization:** Sen Li, Zhichun Wang.

**Writing – original draft:** Sen Li.

**Writing – review & editing:** Yingfang Shi, Zhichun Wang.
